# Impact of Family Sociodemographics and Mother’s Toothbrushing on Australian Preschool Children

**DOI:** 10.1177/23800844251326091

**Published:** 2025-04-03

**Authors:** P. Rajesh, D.H. Ha, L.G. Do, S.K. Tadakamadla

**Affiliations:** 1 School of Medicine and Dentistry, Griffith University, Gold Coast, Australia; 2 School of Dentistry, Faculty of Health and Behavioural Sciences, University of Queensland, Herston, QLD, Australia; 3 Dentistry and Oral Health, Department of Rural Clinical Sciences, La Trobe Rural Health School, La Trobe University, Bendigo, Australia

**Keywords:** preventive dentistry, oral hygiene, dental devices, home care, child, mothers

## Abstract

**Background:**

Understanding the predictors of toothbrushing at the 2 distinct preschool age time points will help develop and implement effective strategies specific to children’s age.

**Aim:**

To examine the effect of a family’s sociodemographic status and mothers’ oral health behavior on children’s toothbrushing frequency at 2 different time points: 2 and 5 y of age.

**Design:**

Secondary analysis (cross-sectional) of longitudinal data collected through a cohort study.

**Results:**

In children aged 2 y, the likelihood of toothbrushing twice or more per day was higher than their comparative counterparts if their mother’s toothbrushing frequency was twice or more per day (odds ratio [OR]: 5.63; 95% confidence interval [CI]: 4.01–7.90), if they were girls (OR: 1.36; 95% CI: 1.04–1.79), if the mother had completed tertiary education (OR: 1.48; 95% CI: 1.01–2.19) or vocational training (OR: 1.54; 95% CI: 1.01–2.33), if the household had 2 adults (OR: 2.48; 95% CI: 1.12–5.50) or 3 or more adults (OR: 2.52; 95% CI: 1.06–5.97), if the total household income was >A$120,000/year (OR: 1.62; 95% CI: 1.03–2.56), if the household had both parents (OR: 2.11; 95 % CI: 1.11–4.02). At the age of 5 y, girls whose mothers brushed their teeth twice or more per day were 1.43 (95 % CI: 1.02–2.02) and 10.53 (95% CI: 7.01–15.80) times more likely to brush their teeth more than twice or more per day than boys whose mothers brushed less than twice per day, respectively.

**Conclusions:**

Child sex and mother’s toothbrushing were the 2 main factors associated with children’s toothbrushing frequency at both ages. In addition, several sociodemographic factors were associated with toothbrushing frequency at 2 y of age.

**Knowledge Transfer Statement:**

The results of this study can be used by parents, especially mothers and policy makers, as they can help promote consistent toothbrushing habits in children. This is crucial as it is a preventive measure against oral health issues and cavities. In addition, the research can play a vital role in shaping policies to improve toothbrushing practices among children between the ages of 2 and 5 y.

**What this article adds:**

- Mothers’ toothbrushing frequency and some family sociodemographic factors were associated with the frequency of toothbrushing in children at 2 y of age. However, at age 5 y, the mother’s toothbrushing frequency and the child’s sex were the only significant variables.

**Why this article is important to pediatric dentists:**

- The article emphasizes the importance of a mother’s toothbrushing practices in determining the toothbrushing frequency of preschool children.
- This article highlights that other sociodemographic factors could also influence toothbrushing frequency among children aged 2 y.

## Introduction

Dental caries is a prevalent chronic disease, significantly affecting disadvantaged groups across all age groups worldwide and is widening disparities in oral health (Schwendicke et al. 2015). The development of dental caries involves multiple factors contributing to demineralization due to microbial activities involving the plaque biofilm (Fejerskov 1997). Studies have also shown that oral diseases in children could influence their ability to chew, drink hot or cold beverages, sleep, and learn (Leong et al. 2013). In some cases, dental caries among children could require treatment under sedation or general anesthesia, which can significantly burden children, their families, and the health care system (Kassebaum et al. 2015; Andrew et al. 2021). Poor oral health in children, therefore, affects children and their families (Quadri et al. 2021). Dental caries is, however, easily preventable by effective preventive measures, both population based and individual strategies, such as brushing with fluoridated toothpaste, community water fluoridation (Do 2020), and better access to dental care (Fisher-Owens et al. 2007). Due to the benefits offered by preventive measures, dental caries have significant public health implications but have received inadequate global attention (Chen et al. 2021). Developing appropriate policies and interventions for ensuring an excellent start to life for all children requires understanding the mechanisms by which certain groups are more prone to practice poor health behaviors and subsequently develop diseases from an early age (Lynch et al. 2010). Therefore, further research is needed to analyze the predictors of oral health outcomes.

Longitudinal studies are likely ideal for studying the ability of behavioral factors, such as toothbrushing patterns, to affect dental caries incidence because toothbrushing frequency varies throughout a child’s life (Foley and Akers 2019). This study uses the data from the Study of Mothers’ and Infants’ Life Events Affecting Oral Health (SMILE) longitudinal study (Do et al. 2020).

The SMILE study collected information at specific age points, including 2 and 5 y of age. While 2 y represents an approximate age for developing toothbrushing behavior with fluoridated toothpaste (Thornton-Evans 2019), children aged 5 y are developing independence while performing toothbrushing (Trinh et al. 2021). Understanding the predictors of toothbrushing at the 2 distinct age time points will help develop and implement effective strategies specific to children’s ages.

**Figure 1:**
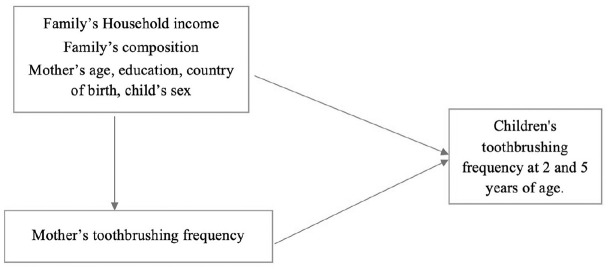
Simplified directed acyclic graph to evaluate the effect of family’s socioeconomic status (SES) and the mother’s toothbrushing frequency on the child’s toothbrushing frequency.

This study aims to evaluate the effect of family sociodemographic factors and mothers’ oral health behaviors on children’s toothbrushing habits at 2 different time points: 2 and 5 y of age (Figure 1).

## Methods

### Study Population and Sampling Methods

In the SMILE study, 2,181 mother–child dyads were recruited at birth between July 2013 and August 2014. Those sufficiently proficient in English to understand the study’s description and instructions, including all new mothers delivering a child in Adelaide’s 3 major maternity hospitals, were invited to participate. A nonprobability convenience sampling method was used to recruit the study population. In the primary study, lower retention rates were expected by people of low socioeconomic backgrounds. Therefore, attempts were made to the baseline to oversample people from low socioeconomic areas to limit the selection bias. The published article provides all the information about the primary study’s methods and data collection process (Do et al. 2020). For the secondary study, permission was obtained from the study’s principal investigators, and the data made available were deidentified to maintain the confidentiality and anonymity of the participants (O’Keefe and Connolly 2010). New ethics clearance was not required because this study involves secondary data analysis.

### Data Collection

At baseline, information was collected from all the participants regarding mother’s age, country of birth, education level, Aboriginal status, work status, health welfare cards, private insurance, mother’s toothbrushing frequency, and the number of adults and children in the household. When the child turned 2 and 5 y of age, additional information, such as the child’s toothbrushing habits, was collected along with the baseline questionnaire. In the primary study, trained staff recruited and collected baseline (wave 1) birth data from mothers through face-to-face interviews. To maximize the retention rate, follow-up data were collected by phone, email, post, and third-party contact. All consenting participants were contacted at each wave, except those who had withdrawn formally, even if they could not complete the previous waves. The data collection time occurred at 4 points in the study’s first phase. The child was assessed at 3 mo (wave 1), 6 mo (wave 2), 1 y (wave 3), and 2 y (wave 4) of age. The data for the second phase were collected from children when they turned 5 y of age (wave 5) and 7 y of age (wave 6).

The data on sociodemographic characteristics were collected at wave 1; the mother’s oral care practices at waves 1, 4, and 5; the child’s oral care practices at waves 3, 4, and 5; and the mother’s self-reported oral health needs and status at waves 3 and 5.

Mother’s education was recoded into 3 categorical variables: high school, vocational training, and tertiary. The household income, which was recorded as 10 categories, was grouped into 3: up to A$60,000, A$60,000 to A$120,000 and more than A$120,000. Household income categories were reported in Australian dollars (A$). For international comparison, the approximate equivalent in US dollars (US$) is also provided, based on the exchange rate at the time of the primary study (1A$ ≈ 0.69 US$): less than A$40,000, approximately US$27,600; A$40,000 to A$80,000, approximately US$27,600 to US$55,200; and greater than A$80,000, approximately US$55,200. Mother’s toothbrushing frequency was categorized into 2 groups: brushing twice or more daily and brushing less than twice daily. The mother’s country of birth was recoded into 2 categories: (1) Australia, New Zealand, and the United Kingdom and (2) other countries. Mother’s age was recategorized into three groups: younger than 24 y, 25 to 35 y, and older than 35 y. Mothers’ Aboriginal status was categorized into Indigenous and non-Indigenous. The family structure included the number of adults in the household, categorized into 1 adult, 2 adults, and 3 or more adults. The number of children was recategorized into single, 2, and 3 or more children. The parent household type had 2 categories: single-parent and 2-parent households. Mother’s relationship was classified into “with a partner” and “without a partner.” Private insurance and health welfare cards were categorized into “those with private insurance and health welfare cards” and “those without either.”

This study’s dependent variable and focus were the children’s toothbrushing frequency, categorized into “brushing twice or more daily” and “brushing less than twice daily” at the ages of 2 and 5 y.

### Statistical Analysis

The data were analyzed using SPSS version 29 (SPSS Inc). Two stages of analysis were adopted. Figure 1 A simplified direct acyclic graph was used to identify the family’s socioeconomic status (SES), and the mother’s toothbrushing frequency was identified as the main exposure for children’s toothbrushing frequency at 2 and 5 y of age. A sequential cross-sectional analysis was conducted using the data from the SMILE cohort study at 2 and 5 y of age to determine whether the family’s sociodemographic status and oral habits influenced the children’s toothbrushing frequency at 2 and 5 y of age. Efforts were made in the primary SMILE study to reduce the attrition and missing data by contacting the participants by phone unless they were officially withdrawn. Despite this effort, there were still missing data. The missing data were addressed and omitted in the analysis methods in this study. We assumed that the missing values were missing at random. Simple and multiple binary logistic regression analysis was used to estimate both unadjusted and adjusted association between the independent and dependent variables; the effect of covariates was controlled to produce an adjusted intervention effect of the family’s sociodemographic status and mother’s toothbrushing frequency on the child’s toothbrushing frequency. The odds ratio and 95% confidence intervals were derived to estimate the association between each independent and dependent variable. A *P* value less than 0.05 was considered statistically significant. We modeled 2 main outcomes: children brushing more than twice or more and brushing less than twice a day. The exposure or predictor factors were the family’s SES and toothbrushing frequency. We used multiple logistic models for binary outcomes to estimate the odds ratio against the reference groups.

## Results

We followed the STROBE checklist throughout the study to ensure comprehensive and transparent reporting. A complete STROBE checklist is included (Appendix Material 1). Data used in the current analysis were sourced at recruitment (birth of the children) and from 2 waves (waves 4 and 5) of SMILE, when the children had turned 2 and 4 y of age, respectively. Table 1 shows the characteristics of the study participants at the baseline and 2 and 5 y of child age. Data collected from the baseline showed that 69.2% of the mothers who participated in the study belonged to the age group of 25 to 35 y, with most mothers (68.1%) born in Australia, New Zealand, and the United Kingdom and being non-Indigenous (97.5%). Approximately half were from the group with a greater than A$60,000 to A$120,000/y total household income (45.3%), had a tertiary education (46.0%), did not have private health insurance (54.6%), and only had 1 child (46.6%). Most children belonged to a 2-adult household (82.0%), and more than half (64.4%) of the mothers brushed their teeth more than twice per day.

**Table 1.** Characteristics of the Study Participants.

	Frequency (%)	Frequency (%)	Frequency (%)
Participant Characteristic	Baseline	2 y of Age	5 y of Age
Mother’s age, y			
<24	348 (16.3)	348 (16.3)	348 (16.3)
25–35 y	1,477 (69.2)	1,477 (69.2)	1,477 (69.2)
>35 y	310 (14.5)	310 (14.5)	310 (14.5)
Mother’s country of birth			
Australia, New Zealand, and the United Kingdom	1,434 (68.1)	1,434 (68.1)	1,434 (68.1)
Other countries	672 (31.9)	672 (31.9)	672 (31.9)
Mother’s Aboriginal status			
Non-Indigenous	2,040 (97.5)	2,040 (97.5)	2,040 (97.5)
Indigenous	53 (2.5)	53 (2.5)	53 (2.5)
Total household income			
≤A$60,000	720 (36.2)	348 (30.6)	164 (19.9)
>A$60,000 to $120,000	902 (45.3)	535 (47.1)	341 (41.4)
>A$120,000	368 (16.9)	254 (22.3)	319 (38.7)
Mother's level of education			
High school	564 (26.8)	564 (26.8)	564 (26.8)
Vocational training	572 (27.2)	572 (27.2)	572 (27.2)
Tertiary	968 (46.0)	968 (46.0)	968 (46.0)
Mother’s relationship status			
With partner	—	—	709 (90.1)
Single/no partner			78 (9.9)
Child’s sex			
Male	1,111 (52.7)	1,111 (52.7)	1,111 (52.7)
Female	997 (47.3)	997 (47.3)	997 (47.3)
Parent household type			
1 parent		151 (12.8)	111 (14.1)
2 parents	—	1,016 (85.9)	674 (30.9)
Health welfare card			
Yes	539 (24.7)	353 (30.4)	189 (24.0)
No or unknown	1,643 (75.3)	810 (69.6)	574 (72.8)
Private insurance other than Medicare			
Yes	942 (45.4)	618 (52.2)	436 (55.3)
No	1,133 (54.6)	565 (47.8)	353 (44.7)
Total number of children in the household			
1 child	965 (46.6)	449 (38.6)	127 (16.2)
2 children	719 (34.8)	495 (42.6)	458 (58.5)
3 or more children	385 (18.6)	219 (18.8)	198 (25.3)
Total number of adults in the household			
1 adult	118 (5.6)	87 (7.5)	76 (9.8)
2 adults	1,722 (82.0)	956 (82.1)	629 (81.1)
3 or more adults	260 (12.4)	121 (10.4)	71 (9.3)
Child’s toothbrushing frequency			
≥2		405 (34.9)	478 (58.2)
<2	—	757 (65.1)	344 (41.8)
Child brushing the night before the bed			
Yes		871 (74.6)	693 (84.4)
No	—	296 (25.4)	128 (15.6)
Mother’s toothbrushing frequency			
≥2	1,353 (64.4)	748 (63.8)	545 (71.1)
<2	748 (35.6)	425 (36.2)	221 (28.9)

— denotes missing data for the category.

Table 2 demonstrates that the mother’s country of birth, Aboriginal status, the total number of children in the household, possession of a health welfare card, or private health insurance were not associated with toothbrushing frequency when children were 2 y old; this was evident from both unadjusted and adjusted analyses. In addition, Tables 2 and 3 show the results from chi-square analysis exploring the association of mother’s SES and tooth practices with children’s toothbrushing frequency with the *P* value.

**Table 2.** Unadjusted and Adjusted Analysis to Predict Mother’s Socioeconomic Status and Toothbrushing Frequency in Australian Children Aged 2 y.

Predictor	Unadjusted Odds Ratio (95% CI)	Adjusted Odds Ratio (95% CI)	χ^2^ (*P* Value)
Mother’s education			
High school	Reference	Reference	
Vocational training	1.24 (0.86–1.79)	**1.54 (1.01–2.33)**	**1.40 (0.50)**
Tertiary	1.08 (0.79–1.49)	**1.48 (1.01–2.19)**	
Mother’s country of birth			
Other countries	Reference	Reference	
Australia, New Zealand, and the United Kingdom	1.01 (0.78–1.31)	1.13 (0.82–1.57)	0.01 (0.94)
Total household income			
≤A$60,000	Reference	Reference	
A$60,000 to A$120,000	0.89 (0.67–1.18)	1.22 (0.84–1.77)	1.06 (0.59)
>A$120,000	1.02 (0.72–1.44)	**1.62 (1.03–2.56)**	
Aboriginal status			
Non-aboriginal	Reference	Reference	
Aboriginal	0.34 (0.76–1.54)	0.24 (0.05–1.18)	
Total number of adults in the household			
1 adult	Reference	Reference	
2 adults	0.98 (0.61–1.55)	**2.48 (1.12–5.50)**	
3 or more adults	1.05 (0.59–1.89)	**2.52 (1.06–5.97)**	
Total number of children in the household			
1 child	Reference	Reference	
2 children	1.00 (0.76–1.30)	1.05 (0.78–1.41)	
3 or more children	1.05 (0.59–1.89)	1.07 (0.73–1.57)	
Health welfare card			
Yes	Reference	Reference	
No	1.23 (0.94–1.60)	1.22 (0.87–1.72)	2.23 (0.14)
Parent household (child’s main place of residence)			
1-parent household	Reference	Reference	
2-parent household	1.34 (0.92–1.95)	2.11 (1.11–4.02)	5.05 (0.08)
Mother’s toothbrushing frequency per day			
<2 times	Reference	Reference	
≥2 times	4.63 (3.43–6.26)	5.63 (4.01–7.90)	109.09 (<0.001)
Sex of the child			
Male	Reference	Reference	
Female	**1.34 (1.05–1.71)**	**1.36 (1.04–1.79)**	**5.67 (0.02)**
Private health insurance			
Yes	Reference	Reference	
No	0.83 (0.65–1.06)	0.93 (0.69–1.24)	2.27 (0.13)

Significant 95% CIs are presented in bold. χ^2^ = 2.34, *P* = 0.97 (Hosmer and Lemeshow test), accuracy = 65.7%, Nagelkerke *R*^2^ = 0.17.

**Table 3.** Unadjusted and Adjusted Analysis to Predict Mother’s Socioeconomic Status and Toothbrushing Frequency in Australian Children Aged 5 y.

Predictor	Unadjusted Odds Ratio	Adjusted Odds Ratio	χ^2^ (*P* Value)
Mother’s education			
High school	Reference	Reference	
Vocational training	1.12 (0.71–1.77)	1.02 (0.58–1.81)	1.40 (**0.50)**
Tertiary	1.46 (0.98–2.16)	0.79 (0.47–1.34)	
Mother’s country of birth			
Other countries	Reference	Reference	
Australia, New Zealand, and the United Kingdom	**1.55 (1.14–2.1)**	1.40 (0.93–2.10)	0.01 (0.94)
Total household income			
≤A$60,000	Reference	Reference	
A$60,000 to A$120,000	1.31 (0.90–1.90)	0.94 (0.52–1.71)	1.06 (0.59)
>A$120,000	**1.78 (1.21–2.61)**	1.29 (0.67–2.5)	
Aboriginal status			
Non-Aboriginal	Reference	Reference	
Aboriginal	2.18 (0.27–21.09)	0.65 (0.06–7.49)	2.15 (0.14)
Total number of adults in the household			
1 adult	Reference	Reference	
2 adults	1.47 (0.91–2.37)	0.63 (0.20–2.00)	0.15 (0.93)
3 or more adults	1.29 (0.67–2.47)	0.70 (0.22–2.22)	
Total number of children in the household			
1 child	Reference	Reference	
2 children	1.11 (0.75–1.66)	0.81 (0.49–1.35)	0.41 (0.81)
3 or more children	1.18 (0.75–1.85)	0.88 (0.50–1.56)	
Health care card			
Yes	Reference	Reference	
No	**1.40 (1.01–1.94)**	1.04 (0.64–1.72)	2.23 (0.14)
Parent household type			
1-parent household	Reference	Reference	
2-parent household	**1.26 (0.84–1.88)**	1.05 (0.39–2.80)	5.05 (0.08)
Mother’s toothbrushing frequency per day			
<2	Reference	Reference	
≥2	**9.64 (6.66–13.95)**	**10.53 (7.01–15.80)**	**109.09 (<0.001)**
Sex of the child			
Male	Reference	Reference	
Female	1.24 (0.94–1.65)	**1.43 (1.02–2.02)**	**5.67 (0.02)**
Private insurance			
No	Reference	Reference	
Yes	1.26 (0.95–1.68)		1.03(0.71–1.50)
Relationship status			
Single/no partner	Reference		Reference
With partner	**1.60 (1.00–2.56)**	1.79 (0.60–5.29)	3.94 (0.05)

Significant 95% CIs are presented in bold. χ^2^ = 5.15, *P* = 0.74 (Hosmer and Lemeshow test), accuracy = 74.7%, *R*^2^ = 0.30.

In the adjusted analysis, the mother’s education, total household income, mother’s toothbrushing frequency, the sex of the child, the household type, and the number of adults in the household were associated with the children’s toothbrushing frequency. Children whose mothers brushed their teeth twice or more a day were more likely to brush them twice or more a day (OR = 5.63; 95% CI = [4.01–7.90]) than those whose mothers brushed less frequently. Girls (OR = 1.36; 95 % CI = [1.04–1.79]) and children belonging to 2-parent households (OR = 2.11; 95% CI = [1.11–4.02]) were more likely to brush their teeth ≥2/d than their comparative counterparts. Furthermore, children from 2-adult households (OR = 2.48; 95% CI = [1.12–5.50]) and 3-adult households (OR = 2.52; 95% CI = [1.06–5.97]) were more likely to brush their teeth ≥2/d than those from single-adult households. Children with mothers who had completed tertiary education (OR = 1.48; 95% CI = [1.01–2.19]) and vocational training (OR = 1.54; 95% CI = [1.01–2.33]) were more likely to brush their teeth ≥2 times a day when compared with mothers with high school education. Children from households with incomes ≥A$120,000 (OR = 1.62; 95% CI = [1.03–2.56]) were more likely to brush their teeth twice or more a day compared with children from families with an income of up to A$60,000.

Table 3 demonstrates that the mother’s education, Aboriginal status, total number of adults in the household, total number of children in the household, private health insurance, and parent household did not significantly contribute to predicting the child’s toothbrushing frequency among Australian children aged 5 y; this was observed in both the unadjusted and adjusted analyses.

In the multivariable analysis, the mother’s toothbrushing frequency and the child’s sex were the only variables significantly contributing to the child’s toothbrushing frequency at 5 y of age. Children whose mothers brushed twice or more a day were more likely to brush their teeth ≥2 times a day than their comparative counterparts were (OR = 10.53; 95% CI = [7.01–15.80]). Girls were more likely to brush their teeth twice or more daily than boys were (OR = 1.43; 95% CI = [1.02–2.02]).

## Discussion

Data related to the oral health behaviors, including toothbrushing frequency, of Australian children younger than 5 y are scarce (Tadakamadla et al. 2022). This study is among the few that have examined the predictors of toothbrushing habits in children aged 2 and 5 y in Australia and other countries. This study found that several family sociodemographic factors and the mother’s toothbrushing frequency were associated with children’s toothbrushing frequency at 2 y of age. However, at 5 y of age, the significant variables were only the mother’s toothbrushing frequency and the child’s gender. A possible explanation for other family sociodemographic factors not being associated with children’s toothbrushing frequency by age 5 y is that most children have established consistent habits and daily routines by age 5. As a result, they might have less reliance on their parents (Wigen and Wang 2015; Berzinski et al. 2020).

Various studies have demonstrated that a mother’s level of education is a significant factor in predicting children’s toothbrushing habits. We have also found that children whose mothers have tertiary or vocational education are more likely to brush their teeth at least twice a day. These findings are consistent with systematic reviews conducted by Hooley et al (2012) and the study among Mexican children by Vallejos-Sánchez et al. (2008). Furthermore, children from higher-income families were more likely to brush their teeth twice a day or more, which aligns with the systematic review by Castilho et al. (2013). As compared with higher-educated mothers and high-income households, less-educated parents may be less aware of the importance of oral hygiene and preventative measures (Cianetti et al. 2017).

Children from 2-adult or 2-parent households were more likely to brush their teeth twice or more a day. This finding is consistent with previous findings from an Australian study that observed that the likelihood of brushing twice daily was reduced by 34% for children living in a single-parent household (Arora et al. 2020). In 2-parent or 2-adult households, parents can minimize child resistance to brushing routines by working together to gain their child’s cooperation and ensuring that both parents consistently emphasize oral health and hygiene to their children (Finlayson et al. 2019).

Mothers who brush their teeth twice or more a day were more likely to brush their children’s teeth twice or more a day. Mothers potentially are positive role models for their children; the study results are consistent with previous studies demonstrating that maternal oral health behavior (toothbrushing) is associated with favorable toothbrushing behavior in children (Mohebbi et al. 2008; Olak et al. 2018).

In this study, the mother’s toothbrushing frequency was a significant predictor of children’s toothbrushing frequency at both ages. This demonstrates that consistent behavioral habits are learned at home, with parents, especially the primary caretaker, modeling the expected behavior (Aunger 2007). During the early years of life, children learn good oral hygiene habits that are deeply ingrained in their minds, which should lead to better oral hygiene later in life (Pullishery et al. 2013).

In addition, the study found girls to be more likely to brush their teeth twice or more daily as compared with boys at both the 2- and 5-y time points. Research has shown that girls tend to more frequently comply with adult requests at a younger age than boys do (Feingold 1994).

Another meta-analysis found that the girls displayed more prosocial behaviors than the boys did when an adult was present (Fabes and Eisenberg 1998). Such gender differences can be attributed to girls being more compliant with their parent’s instructions to maintain good oral hygiene.

This study’s potential limitation is that it used secondary data, which may have led to residual confounding in the secondary analysis. Despite this potential limitation, the study thoroughly investigated the factors predicting children’s toothbrushing. The study design was longitudinal and one of the largest, with a significant sample size that provided ample statistical power. This study’s findings may be limited in generalizability as they were conducted in a specific setting: the primary SMILE study in Adelaide, South Australia. While the study provides valuable insights into the factors affecting oral health in young children, its results are most relevant to similar populations within Australia. Caution should be exercised when attempting to generalize the findings to other regions or countries, as differences in socioeconomic, cultural, and health care contexts could influence the outcomes.

### Future Implications

While acknowledging that pregnant women often concentrate on immediate concerns related to childbirth, pregnancy also offers a unique opportunity to promote preventive health behaviors through routine antenatal care. By improving oral health literacy during this time, we can achieve a dual benefit: encouraging better oral health practices among mothers and positively affecting their children’s oral health outcomes. This strategy could support broader initiatives to tackle socioeconomic disparities, as maternal oral health knowledge plays a vital role in shaping family oral health behaviors. Future research should investigate the incorporation of oral health education into prenatal care as a standard practice to maximize its potential impact.

## Conclusion

To summarize, the study found that the influence of family sociodemographic factors on toothbrushing frequency was more noticeable in 2-y-old children than in 5-y-old children. Also, the mother’s toothbrushing frequency was consistently associated with children’s toothbrushing frequency. As such, emphasizing the crucial role of a mother’s toothbrushing habits in promoting good oral hygiene in children is of utmost importance.

## Author Contributions

P. Rajesh, contributed to analysis and interpretation, drafted the manuscript; D.H. Ha and L.G. Do contributed to the conception, design, data acquisition, analysis, and interpretation and critically revised the manuscript; S.K. Tadakamadla, contributed to analysis and interpretation, critically revised the manuscript. All authors gave final approval and agree to be accountable for all aspects of the work.

## Supplemental Material

sj-pdf-1-jct-10.1177_23800844251326091 – Supplemental material for Impact of Family Sociodemographics and Mother’s Toothbrushing on Australian Preschool Children Supplemental material, sj-pdf-1-jct-10.1177_23800844251326091 for Impact of Family Sociodemographics and Mother’s Toothbrushing on Australian Preschool Children by P. Rajesh, D. Ha, L.G. Do and S.K. Tadakamadla in JDR Clinical & Translational Research

## References

[bibr1-23800844251326091] Andrew L Wallace R Wickens N Patel J. 2021. Early childhood caries, primary caregiver oral health knowledge and behaviours and associated sociological factors in Australia: a systematic scoping review. BMC Oral Health. 21(1):521. 10.1186/s12903-021-01887-4. 34645446 PMC8513214

[bibr2-23800844251326091] Arora A Nargundkar S Fahey P Joshua H John JR. 2020. Social determinants and behavioural factors influencing toothbrushing frequency among primary school children in rural Australian community of Lithgow, New South Wales. BMC Res Notes. 13(1):403. 10.1186/s13104-020-05239-3. 32859256 PMC7456049

[bibr3-23800844251326091] Aunger R . 2007. Tooth brushing as routine behaviour. Int Dent J. 57(suppl 5):364–376.

[bibr4-23800844251326091] Berzinski M Morawska A Mitchell AE Baker S. 2020. Parenting and child behaviour as predictors of toothbrushing difficulties in young children. Int J Paediatr Dent. 30(1):75–84. 31408252 10.1111/ipd.12570

[bibr5-23800844251326091] Castilho AR Mialhe FL Barbosa TdS Puppin-Rontani RM . 2013. Influence of family environment on children’s oral health: a systematic review. J Pediatr (Rio J). 89(2):116–123. 23642420 10.1016/j.jped.2013.03.014

[bibr6-23800844251326091] Chen J Duangthip D Gao SS Huang F Anthonappa R Oliveira BH Turton B Durward C El Tantawi M Attia D , et al. 2021. Oral health policies to tackle the burden of early childhood caries: a review of 14 countries/regions. Front Oral Health. 2:670154. 10.3389/froh.2021.670154. 35048013 PMC8757786

[bibr7-23800844251326091] Cianetti S Lombardo G Lupatelli E Rossi G Abraha I Pagano S Paglia L. 2017. Dental caries, parents educational level, family income and dental service attendance among children in Italy. Eur J Paediatr Dent. 18(1):15–18. 28494596 10.23804/ejpd.2017.18.01.03

[bibr8-23800844251326091] Do LG , Australian Research Centre for Population Oral Health. 2020. Guidelines for use of fluorides in Australia: update 2019. Aust Dent J. 65(1):30–38. 31868926 10.1111/adj.12742

[bibr9-23800844251326091] Do LG Ha DH Bell LK Devenish G Golley RK Leary SD Manton DJ Thomson WM Scott JA Spencer AJ . 2020. Study of Mothers’ and Infants’ Life Events affecting oral health (SMILE) birth cohort study: cohort profile. BMJ Open. 10(10):e041185. 10.1136/bmjopen-2020-041185. PMC7590353 33099500

[bibr10-23800844251326091] Fabes RA Eisenberg N. 1998. Meta-analyses of age and sex differences in children’s and adolescents’ prosocial behavior. Tempe (AZ): Arizona State University; [accessed 2025 Feb 19]. https://www.public.asu.edu/~rafabes/meta.pdf.

[bibr11-23800844251326091] Feingold A . 1994. Gender differences in personality: a meta-analysis. Psychol Bull. 116(3):429–456. 7809307 10.1037/0033-2909.116.3.429

[bibr12-23800844251326091] Fejerskov O . 1997. Concepts of dental caries and their consequences for understanding the disease. Community Dent Oral Epidemiol. 25(1):5–12. 9088687 10.1111/j.1600-0528.1997.tb00894.x

[bibr13-23800844251326091] Finlayson TL Cabudol M Liu JX Garza JR Gansky SA Ramos-Gomez F. 2019. A qualitative study of the multi-level influences on oral hygiene practices for young children in an Early Head Start program. BMC Oral Health. 19(1):166. 10.1186/s12903-019-0857-7. 31349826 PMC6660967

[bibr14-23800844251326091] Fisher-Owens SA Gansky SA Platt LJ Weintraub JA Soobader M-J Bramlett MD Newacheck PW . 2007. Influences on children’s oral health: a conceptual model. Pediatrics. 120(3):e510–e520. 10.1542/peds.2006-3084. 17766495

[bibr15-23800844251326091] Foley M Akers HF . 2019. Does poverty cause dental caries? Aust Dent J. 64(1):96–102. 30444538 10.1111/adj.12666

[bibr16-23800844251326091] Hooley M Skouteris H Boganin C Satur J Kilpatrick N. 2012. Parental influence and the development of dental caries in children aged 0–6 years: a systematic review of the literature. J Dent. 40(11):873–885. 22842202 10.1016/j.jdent.2012.07.013

[bibr17-23800844251326091] Kassebaum NJ Bernabé E Dahiya M Bhandari B Murray CJL Marcenes W. 2015. Global burden of untreated caries: a systematic review and metaregression. J Dent Res. 94(5):650–658. 25740856 10.1177/0022034515573272

[bibr18-23800844251326091] Leong PM Gussy MG Barrow SYL de Silva-Sanigorski A Waters E. 2013. A systematic review of risk factors during first year of life for early childhood caries. Int J Paediatr Dent. 23(4):235–250. 22925469 10.1111/j.1365-263X.2012.01260.x

[bibr19-23800844251326091] Lynch JW Law C Brinkman S Chittleborough C Sawyer M . 2010. Inequalities in child healthy development: some challenges for effective implementation. Soc Sci Med. 71(7):1244–1248. 20691527 10.1016/j.socscimed.2010.07.008

[bibr20-23800844251326091] Mohebbi SZ Virtanen JI Murtomaa H Vahid-Golpayegani M Vehkalahti MM . 2008. Mothers as facilitators of oral hygiene in early childhood. Int J Paediatr Dent. 18(1):48–55. 18086026 10.1111/j.1365-263X.2007.00861.x

[bibr21-23800844251326091] O’Keefe CM Connolly CJ . 2010. Privacy and the use of health data for research. Med J Aust. 193(9):537–541. 21034389 10.5694/j.1326-5377.2010.tb04041.x

[bibr22-23800844251326091] Olak J Nguyen MS Nguyen TT Nguyen BBT Saag M. 2018. The influence of mothers’ oral health behaviour and perception thereof on the dental health of their children. EPMA J. 9(2):187–193. 29896317 10.1007/s13167-018-0134-x PMC5972135

[bibr23-23800844251326091] Pullishery F Shenoy Panchmal G Shenoy R. 2013. Parental attitudes and tooth brushing habits in preschool children in Mangalore, Karnataka: a cross-sectional study. Int J Clin Pediatr Dent. 6(3):156–160. 25206214 10.5005/jp-journals-10005-1210 PMC4086598

[bibr24-23800844251326091] Quadri MFA Jaafari FRM Mathmi NAA Huraysi NHF Nayeem M Jessani A Tadakamadla SK Tadakamadla J . 2021. Impact of the poor oral health status of children on their families: an analytical cross-sectional study. Children (Basel). 8(7):586. 10.3390/children8070586. 34356565 PMC8305805

[bibr25-23800844251326091] Schwendicke F Dörfer C Schlattmann P Page LF Thomson W Paris S. 2015. Socioeconomic inequality and caries: a systematic review and meta-analysis. J Dent Res. 94(1):10–18. 25394849 10.1177/0022034514557546

[bibr26-23800844251326091] Tadakamadla SK Rathore V Mitchell AE Kaul A Morawska A. 2022. Child- and family-level factors associated with toothbrushing frequency in a sample of Australian children. Int J Paediatr Dent. 32(5):639–648. 34811821 10.1111/ipd.12942

[bibr27-23800844251326091] Thornton-Evans G . 2019. Use of toothpaste and toothbrushing patterns among children and adolescents—United States, 2013–2016. MMWR Morb Mortal Wkly Rep. 68(4):87–90. 30703075 10.15585/mmwr.mm6804a3 PMC6400578

[bibr28-23800844251326091] Trinh VA Tarbit E Do L Ha D Tadakamadla SK . 2021. The influence of family socioeconomic status on toothbrushing practices in Australian children. J Public Health Dent. 81(4):308–315. 34622451 10.1111/jphd.12477

[bibr29-23800844251326091] Vallejos-Sánchez AA Medina-Solís CE Maupomé G Casanova-Rosado JF Minaya-Sánchez M Villalobos-Rodelo JJ Pontigo-Loyola AP . 2008. Sociobehavioral factors influencing toothbrushing frequency among schoolchildren. J Am Dent Assoc. 139(6):743–749. 18519998 10.14219/jada.archive.2008.0256

[bibr30-23800844251326091] Wigen TI Wang NJ . 2015. Does early establishment of favorable oral health behavior influence caries experience at age 5 years? Acta Odontol Scand. 73(3):182–187. 25385683 10.3109/00016357.2014.976264 PMC4692963

